# High-Energy Excimer Annealing of Nanodiamond Layers

**DOI:** 10.3390/nano13030557

**Published:** 2023-01-30

**Authors:** Klaudia Hurtuková, Nikola Slepičková Kasálková, Dominik Fajstavr, Ladislav Lapčák, Václav Švorčík, Petr Slepička

**Affiliations:** 1 Department of Solid State Engineering, University of Chemistry and Technology Prague, 166 28 Prague, Czech Republic; 2 Central Laboratories, University of Chemistry and Technology, 166 28 Prague, Czech Republic

**Keywords:** carbon, excimer laser, Q-carbon, nanodiamonds, raman spectroscopy, nanostructure, surface analysis

## Abstract

Here, we aimed to achieve exposure of a nanodiamond layer to a high-energy excimer laser. The treatment was realized in high-vacuum conditions. The carbon, in the form of nanodiamonds (NDs), underwent high-temperature changes. The induced changes in carbon form were studied with Raman spectroscopy, X-ray photoelectron spectroscopy, and X-ray diffraction (XRD) and we searched for the Q-carbon phase in the prepared structure. Surface morphology changes were detected by atomic force microscopy (AFM) and scanning electron microscopy (SEM). NDs were exposed to different laser energy values, from 1600 to 3000 mJ cm^−2^. Using the AFM and SEM methods, we found that the NDs layer was disrupted with increasing beam energy, to create a fibrous structure resembling Q-carbon fibers. Layered micro-/nano-spheres, representing the role of diamonds, were created at the junction of the fibers. A Q-carbon structure (fibers) consisting of 80% sp^3^ hybridization was prepared by melting and quenching the nanodiamond film. Higher energy values of the laser beam (2000 and 3000 mJ cm^−2^), in addition to oxygen bonds, also induced carbide bonds characteristic of Q-carbon. Raman spectroscopy confirmed the presence of a diamond (sp^3^) phase and a low-intensity graphitic (G) peak occurring in the Q-carbon form samples.

## 1. Introduction

Carbon is a widespread and well-known natural element, it has the ability to occur in several different structural forms, which often have significantly different properties. Graphite and diamond are the two basic carbon materials found in nature [1]. Thanks to the hybridization of sp, sp^2^, and sp^3^ orbitals, other forms of carbon have been discovered, such as fullerene [2], carbon nanotubes [3] and nanofibers [4], and graphene [5,6,7,8]. In recent years, the discovery of a new carbon phase called Q-carbon (“quenched” carbon) has attracted significant attention. Behind this discovery is a group of scientists headed by Narayan, who described its structure as formed by a unique combination of about 80% sp^3^ hybridization, the rest being sp^2^ hybridization [9]. Narayan et al. described the properties of Q-carbon itself as exceptional. Compared to diamond, Q-carbon has up to 40% more hardness, excellent superconductivity at high temperatures, an extraordinary Hall effect [10], chemical inertness, and abrasion resistance [11]. Q-carbon has been also reported as a new radiation-resistant material [12].

Q-carbon is created by nanosecond laser melting of amorphous carbon followed by ultra-fast cooling, a process called ”quenching” [13]. It has been shown that nanosecond laser heating of diamond-like carbon (DLC) on sapphire, glass, and polymer substrates can lead to reduced carbon melting in the supercooled state [14]. Amorphous carbon films, also known as diamond-like carbon (DLC) coating, are most often prepared through pulsed vapor deposition (PVD) methods or a combination of PVD and chemical vapor deposition (CVD) techniques [15]. The CVD method produces thin films by chemical reaction of precursors in the gas phase on a heated substrate. Unlike PVD methods, such as evaporation and sputtering, CVD offers some advantages by relying on chemical reactions that allow tunable deposition rates, and yields high-quality products with excellent conformability [16]. The pulsed laser deposition (PLD) technique is a type of PVD [17,18,19] that differs from other PVD methods (mentioned above) in two significant ways. First, a high-power pulsed laser is used for synthesis, which means that the thickness of the deposited material (10–500 nm) [17] can grow within a few microseconds. Second, due to the rapid, intense heating of the target, stoichiometric growth can easily be achieved. Compared to the CVD technique, the PLD method is simple, versatile, fast [20], and cost-effective. It allows for the good control of thickness and morphology, requires low temperatures for growth, and can be used with temperature-sensitive materials, especially those with an active chemical surface [18].

During the quenching process, and the overall production of Q-carbon, various carbon polymorphs are created as side products [21]. Nanodiamonds, the newest member of the carbon nanoparticle family, with an average size of 5 nm, have attracted enormous attention due to their distinct physical and chemical properties. A diamond core in the structure of nanodiamonds provides a chemically inert core, high thermal conductivity, and hardness [22,23]. Nanodiamonds are also characterized by a low toxicity, excellent biocompatibility [24], and a large surface area, which is particularly important in the pharmacy field for drug transport or targeted delivery to specific tissues/organs [22]. Nanodiamonds also have fluorescence capabilities that can be used in the imaging field as imaging probes [23]. The side product group also includes diamond thin films, one of the most fascinating and universal materials [10] for various technological applications of great commercial importance [25]. Depositing a diamond film on the surface of a single-crystal sapphire is in great demand mainly because of its properties, which are optical transparency, high melting point, high hardness, chemical inertness, and many others [25,26]. Applications of diamond film on sapphire include diamond polishing wheels, corrosion- and radiation-resistant infrared windows, solid-state lasers, and new scanning probe microscopy applications, such as nanoindentation and high-resolution imaging of soft samples [25].

After cooling the melt, an obvious Q-carbon/α-carbon interface is formed, where α-carbon fulfills the function of amorphous carbon with a high content of sp^2^ hybridization (≈60%) [27]. The name “alpha” carbon was obtained from the amorphous phase formed after cooling. It also distinguishes this form from the amorphous phase of Q-carbon and DLC [28]. This type of carbon (α-carbon), in combination with other phases, increases the resulting composite material’s toughness and provides energy absorption, which is of considerable importance for applications in the machine tools industry [21,29]. Thanks to the CVD technique, it could be feasible to deposit diamonds on the surface of various substrates and thus improve their properties. One of the primary targets of CVD diamond applications is the coating of cutting machines [30,31]. These are widely used for ultra-precision chemical machining due to their excellent wear resistance, sharp cutting edge, low affinity, and other desirable properties. Even though diamond is considered the hardest material worldwide, diamond-cutting tools are still subject to wear after prolonged use or when working with hard materials. This subsequently reduces the machined surface’s quality and the shape’s accuracy. This is why the industry strives to improve diamond-cutting machines’ quality and efficiency [32,33,34], or are even applied to carbide tools [35].

In this study, we modified the nanocrystalline diamond film (NDs) using a laser excimer beam (1600, 2000, and 3000 mJ cm^−2^), and investigated its effect on the change in NDs surface morphology and chemical composition. The surface’s transformation was studied using atomic force microscopy (AFM) and scanning electron microscopy (SEM). Energy-dispersive spectroscopy (EDS), X-ray photoelectron spectroscopy (XPS), Raman spectroscopy, and X-ray diffraction (XRD), these methods were used to observe the changes in the surface’s chemical composition and its binding relationships leading to Q-carbon formation.

## 2. Materials and Methods

### 2.1. Materials

We used commercially available ultrananocrystalline diamond film (1000 nm thick film, grain size: 200–300 nm, supplied by Goodfellow Ltd., Huntingdon, UK) prepared by a chemical vapor deposition (CVD) process on a high-purity silicon wafer. The surface of the nanodiamond (ND) film was modified with a high-energy pulsed excimer KrF laser (Coherent Inc., Santa Clara, CA, USA, Leap 100 K) with a wavelength of 248 nm and a pulse duration of 20–40 ns. The repetition rate of 1 Hz, with output above 1000 mJ, allowed us to expose an area of 32 × 13 mm^2^ with a single laser shot. An aperture of 30 × 10 mm^2^ was used in the experiments, with the application of a lens with the ability to increase the laser fluence up to 3000 mJ cm^−2^.

### 2.2. Analytical Methods 

A detailed analysis of the surface morphology and elemental composition was acquired with a scanning electron microscope (SEM) and energy-dispersive X-ray spectroscopy (EDS). We used a scanning electron microscope, the LYRA3 GMU (Tescan, Brno, Czech Republic) with an applied acceleration voltage of 10 kV for the bombarded electrons, and an F-MaxN analyzer and SDD detector (Oxford Instruments, Abingdon, UK) with an applied acceleration voltage of 10 kV for EDS. 

Atomic force microscopy (AFM) was used to study the surface morphology and roughness of modified NDs film via the high-power excimer laser. The movement of the tip as it passed over the sample was recorded, and a point-by-point image of the surface was compiled. For AFM analysis, we used Dimension ICON (Bruker Corp., Billerica, MA, USA), and ScanAsyst mode in the air was used for determination. A silicon tip on the nitride lever, SCANASYST-AIR, with an elasticity constant of 0.4 N m^−1^, was used. The NanoScope Analysis software (version 1.80, Bruker Corp., Billerica, MA, USA) was applied for data processing. The mean roughness value (Ra) represents the average of the deviations from the center plane of the sample. 

The presence and oxidation state of carbon, and the partial presence of oxygen, in the surface layer was determined by X-ray photoelectron spectroscopy (XPS). An Omicron Nanotechnology ESCAProbeP spectrometer (Omicron nanotechnology GmbH, Taunusstein, Germany) was used. The exposed and analyzed area had a dimension of 2 × 3 mm^2^. The X-ray source was monochromatic at 1486.7 eV. Atomic concentrations of elements were determined by the CASA XPS program using an integrated area of spectrum lines quoted in the database of CASA XPS. The samples were analyzed under a take-off angle of 19°.

Raman analysis was performed on a dispersed Raman spectrometer DXR Microscope from Thermo Scientific (Waltham, MA, USA), equipped with a confocal microscope Olympus and thermoelectrically cooled CCD detector. As an excitation source, we used a solid-state Nd:YAG laser (wavelength 532 nm, maximum power 10 mW). A grating with 900 lines/mm, 25 µm slit aperture, and 50× magnification objective was utilized. Measurement conditions for the samples were 10 mW laser power, 10 s acquisition time per scan, and 20 repetitions. Ten spectra were averaged from each surface. Data were processed using the Omnic 9 software (Thermo Scientific, Waltham, MA, USA).

The diffraction pattern for the material was collected at room temperature with an X’Pert3 Powder θ-θ powder diffractometer with parafocusing Bragg-Brentano geometry using Cu Kα radiation (λ = 1.5418 Å, Ni filter, generator setting: 40 kV, 30 mA). An ultrafast PIXCEL detector with 255 channels was employed to collect XRD data over the angular range from 40 to 50° 2θ, with a step size of 0.039° 2θ and a counting time of 3.9 s/step.

## 3. Results

### 3.1. Surface Morphology Using SEM and AFM Methods

**Figure 1:**
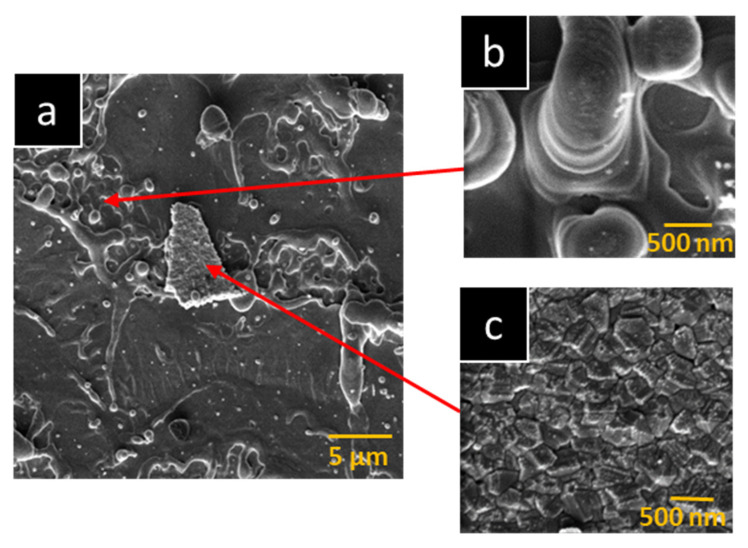
Scanning electron microscopy images of NDs film exposed to high-energy excimer laser fluence for 3000 mJ cm^−2^. The scanning areas of the hybrid area (**a**) were 30 × 30 µm^2^, and scans for fibrous structure with micro-/nano-balls (**b**) and original sharply defined structure of NDs (**c**) were 3 × 3 µm^2^.

In this study, we focused on the possibility of remelting a nanodiamond film to obtain a new form of carbon called Q-carbon. As already mentioned in the Introduction, Q-carbon is prepared based on the principle of nanosecond laser-melting of amorphous carbon. At the same time, essential by-products, NDs, are formed. The idea of directly converting prepared NDs into Q-carbon, while eliminating the creation of amorphous carbon via the PLD method, was designed. Our experiment used a silicon substrate coated with a film of ultrananocrystalline 200–300 nm nanodiamonds prepared via the CVD method [36]. Since NDs have strong C-C bonds [37], it was necessary to use a pressure chamber and high-energy values of the laser to disrupt them and create a new phase—in our case, the fluence 1600, 2000, and 3000 mJ cm^−2^ was applied. The surface morphology change after the laser beam application was investigated using the SEM method and the AFM method. Exposing the NDs film to high energies induced a strong stress in the layer, which caused the structure to tear, and led to partial remelting of the NDs. Figure 1 shows SEM images of the created hybrid surface (Figure 1a) after exposure to the beam (3000 mJ cm^−2^), together with zoomed-in images of the given areas (Figure 1b,c). As can be seen, after the pulse annealing of the NDs film, the original sharply defined icosahedral structure of the NDs (Figure 1c) [38] was transformed into a fibrous structure formed by layered circular micro-/nano-spheres (Figure 1b).

The preparation of a fibrous structure by quenching a supercooled carbon layer has already been described many times by scientists in several professional articles [14,39]. These research papers proceeded from the assumption that interphase instability occurs at the film–substrate interface, caused by compressive stress. This subsequently evokes tensile stress in the top layer, causing it to crack and expose the Q-carbon as a fibrous structure. 

**Figure 2:**
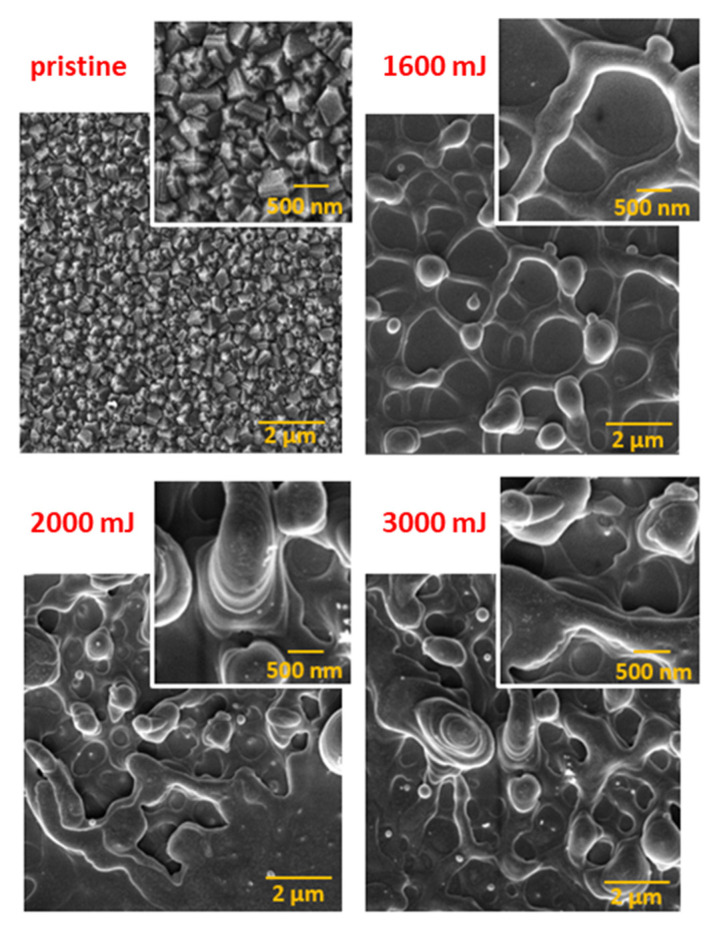
Scanning electron microscopy images of an unmodified (pristine) NDs film, and an NDs film exposed to high-energy excimer laser and laser fluencies for 1600, 2000, and 3000 mJ cm^−2^. The scanning areas were 10 × 10 µm^2^ (main picture) and 3 × 3 µm^2^ (corner picture).

According to [9], the Q-carbon fiber’s diameter was 200–500 nm, which corresponds to the fiber width shown in Figure 2. Figure 2 compares SEM images of the modified NDs film surface after exposure to different beam energy values (1600, 2000, and 3000 mJ cm^−2^). As mentioned above, we prepared micro-/nano-spheres on its surface, in addition to the fibrous structure. The formation of balls could be caused by various factors that also affect their functions. The spheres can behave, for example, as microdiamonds, created either by melting and back-clustering of NDs or as a by-product in the preparation of Q-carbon. They can also behave like “Q-carbon nanoballs”, that coagulate with each other and form larger clusters along the chains [40]. The size of the balls depends on the growth rate and the time. The authors of [41] state that the absence of a nucleation barrier in Q-carbon may also be responsible for the resulting size of the nanospheres. In our case, the change in the fibrous structure’s shape and the balls’ size was also influenced by the laser-annealing energy, as seen in Figure 2. Taking a closer look at the microstructures, it is seen that when the laser energy was reduced, the number and size of the balls decreased, and the overall layering of the structures also decreased. This may be caused by the poor melting of the NDs film, and therefore the most visible demarcation of the fibrous structure with distinct micro-/nano-spheres can be seen on the sample using the lowest energy of 1600 mJ cm^−2^. 

**Figure 3:**
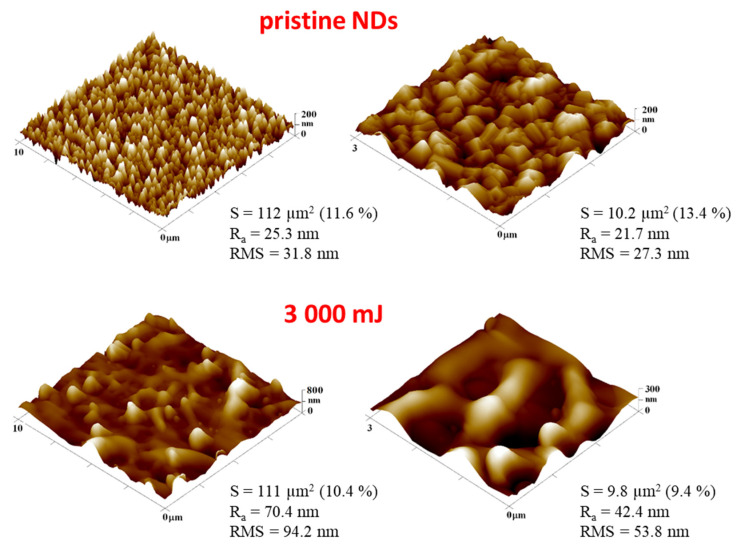
Atomic force microscopy images of pristine NDs and NDs exposed to high-energy excimer laser and laser fluencies for 3000 mJ cm^−2^. The scanning areas were 10 × 10 µm^2^ (**left**), and the detail of the morphology was 3 × 3 µm^2^ (**right**). R_a_ represents the average surface roughness, and RMS represents the root mean square roughness, integral area (S), and the difference from the basic area.

Another method used for investigating changes in surface morphology produced by laser beam exposure was atomic force microscopy (AFM). In Figure 3, we can see the change from the NDs structure with sharp edges (pristine), to a structure composed of micro-/nano spheres with intertwined fibers formed after laser application at an energy of 3000 mJ cm^−2^. As with the images obtained by the SEM method, in the case of AFM imaging, it is possible to reshape the pristine NDs film using sufficiently high energy. Due to the increased fluence of the laser, there was a significant change in the roughness of the surface. High roughness values corresponded to higher peaks in the structure.

### 3.2. Surface Chemistry Measured Using EDS and XPS Methods 

**Figure 4:**
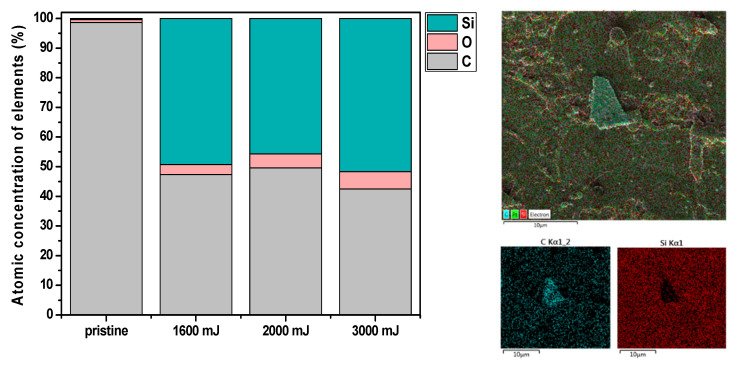
The atomic concentrations of C, O, and Si elements (in wt %) were determined using the energy-dispersive X-ray spectroscopy method for the pristine NDs and NDs film exposed to high-energy excimer laser and laser fluencies for 1600, 2000, and 3000 mJ cm^−2^. The figure contains an EDS map of the distribution of elements (C, Si) on the surface of the sample after a laser pulse of 3000 mJ cm^−2^.

Changes in the chemical composition of the elements of the NDs film modified by laser annealing were analyzed using the EDS method. As mentioned in Section 3.1, the quenching of NDs leads to the film’s destruction (“tearing”) and the creation of new fibrous structures. This causes a change in the total height of the NDs layer and a change in the chemical composition. Here, it is important to note that the EDS method measures the chemical composition of elements to a certain depth, which can detect the presence of elements other than those on the surface. For example, this phenomenon can be observed in a sample of NDs without laser modification (pristine NDs). Figure 4 shows a graph describing the different element concentrations of the samples depending on the different energy of a single laser shot, and the EDS map of the distribution of elements on the sample surface after a laser pulse of 3000 mJ cm^−2^. Since the NDs film is coated on a silicon substrate, during the chemical analysis of pristine NDs, in addition to carbon (98.6%), silicon (0.4%) and oxygen (1.0%) were also present in the form of SiO_2_ (see the graph in Figure 4). It can be seen from the graph that laser annealing rapidly increases the concentration of Si in all samples. The reason for this phenomenon may be the partial blasting of the NDs film and, thus, the depth measurement of EDS. Another possibility could be the deposition of silicon and oxygen from the atmosphere after laser deposition, and exposure of the sample to ambient air.

However, it is necessary to mention that high-energy electrons excite X-rays to a certain depth of the sample (up to 3 µm), but these rays have an escape depth that is much deeper. The main issue applies if an electron beam perpendicular to the surface is used. Here, a problem can arise when analyzing particles smaller than 0.2 µm on a SiO_2_ film, as most of the signal can be generated from the underlying film, and these particles are incorrectly identified as SiO_2_ [42].

**Figure 5:**
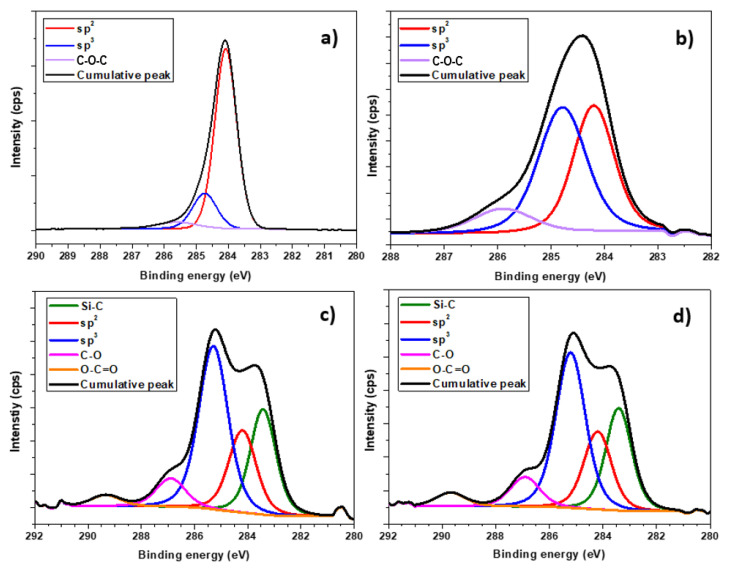
Detailed XPS spectra of the C1s band (**a**) pristine NDs sample and samples with deposited laser energy, (**b**) 1600 mJ cm^−2^, (**c**) 2000 mJ cm^−2^, (**d**) 3000 mJ cm^−2^.

For a more detailed view of the binding characteristics of the samples, the XPS method was used, which measures the kinetic energy of electrons from the upper ten atomic layers [43]. Figure 5 shows XPS C 1 s spectra of pristine NDs and samples exposed to a single laser shot (1600, 2000, and 3000 mJ cm^−2^). The deconvolved bonds observed at 283.4, 284.1–284.2, 284.7–285.3, 286.9, and 289.3–289.7 eV are typical for Si-C bonds, sp^2^-hybridized carbon bonds, sp3-hybridized carbon bonds, C-O bonds, and O-C=O bonds. In the samples of 1600 mJ and pristine NDs, we can observe a peak in the range of 285.6–286.0 eV, attributed to the C-O-C bond [44]. A great surprise during the spectral analysis was that sp^2^ hybridization occurred in an enormous proportion of up to 78% in the sample of pristine NDs (see Figure 5). One of the reasons for this unusual composition may be the processing of the overall resulting film. According to [45], the carbon sp^3^ and sp^2^ hybridization ratio depends on the film deposition conditions, and can also vary from pure diamond to pure graphite. Exposure of the NDs surface to a laser beam (1600, 2000, and 3000 mJ cm^−2^) led to an increase in sp^3^ carbon hybridization, surface oxidation, and the formation of Si-C bonds, at the expense of a decrease in sp^2^ carbon bonds. Their conversion to diamond sp^3^ bonds can cause a decrease in the intensity of sp^2^ bonds. The authors of [44] reported that bombarding an NDs film with sufficiently high ion energy could generate Frenkel pairs, that change the sp^2^ to sp^3^ bonds required for the Q-carbon structure. The presence of oxygen in the 2000 mJ cm^−2^ and 3000 mJ cm^−2^ samples could be caused by the destruction of bonds (the formation of a reaction surface) in the NDs film when oxygen from the surrounding atmosphere was bound after it was removed from the vacuum chamber. A peak in the region of 283.4 eV, corresponding to the Si-C bond, also appeared in these samples. This connection could have occurred due to the high deformation of the so-called partial blasting of the NDs film prepared on a silicon substrate. The percentages of each bond in the pristine NDs samples and the samples irradiated with the laser beam (1600 mJ cm^−2^, 2000 mJ cm^−2^, and 3000 mJ cm^−2^) are shown in Table 1.

### 3.3. Raman Spectroscopy

**Figure 6:**
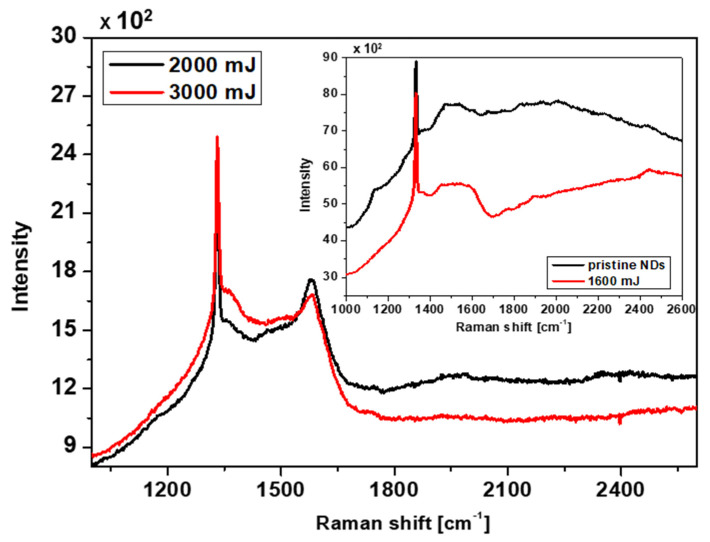
Raman spectra of pristine NDs and 1600 mJ cm^−2^ samples (corner spectrum), and 2000 mJ cm^−2^ and 3000 mJ cm^−2^ samples (main spectrum).

The bonding characteristics of the samples prepared by a single excimer laser shot were studied using Raman spectroscopy, describing the bonding relationships in different structures. The Raman spectra of samples treated with an excimer laser pulse can be divided into two groups according to the position of the deconvolution peaks. Figure 6 shows the spectra of samples with deposition energies of 2000 and 3000 mJ cm^−2^ (primary spectrum), and a corner spectrum representing the pristine NDs sample with a sample of 1600 mJ cm^−2^. As is generally known, the Raman spectra of these structures are characterized by two significant peaks: the D peak, reflecting the band of defects, and the G peak, characterizing untransformed graphite. According to [45], diamond films prepared mainly by the CVD method consist of many small micro-/nano-crystalline diamonds surrounded by a graphitic or amorphous carbon phase mainly occurring at their grain boundaries. Due to the presence of these phases, C-H bonds and C-C and C=C transpolyacetal chains are bent/stretched, which causes the appearance of a Raman peak in the region of 1140 cm^−1^ [46,47] (see the NDs pattern in Figure 6). Additionally, the authors of [45] state that the graphitic (G) peak observed in NDs films prepared in this way was shifted from the graphitic peak of 1580 cm^−1^ downwards to a broad band in the region of 1500 to 1600 cm^−1^.

In our case, this statement agrees with the XPS spectrum of the pristine NDs sample in Figure 5, which consists mainly of sp^2^ hybridized carbon. On the other hand, the D band characterizing various modifications in the film structure may not be visible in the Raman spectrum [45]. The 1600 mJ cm^−2^ sample features very similar deconvoluted peaks to the pristine NDs sample. This is due to the weak deposition energy of the laser beam on the surface of the NDs, where no significant change in binding interactions occurred. For all spectra in Figure 6, we can see a distinct peak in the region of 1332 cm^−1^, which belongs to sp^3^ diamond hybridization or samples containing the diamond phase [48,49]. In the case of the 2000 mJ cm^−2^ and 3000 mJ cm^−2^ samples, it can be either the remains of the NDs film or the micro-/nano-diamonds formed at the triple points of the intersecting Q-carbon fibrous structure shown in the SEM images in Figure 2. This statement is also confirmed by the XPS spectra shown in Figure 5, where a significant conversion of sp^2^ to sp^3^ hybridization can be seen. Since Q-carbon consists of 80% sp^3^ hybridization, it is assumed that the fibrous structures that were created contain a Q-carbon phase [50]. There is also a prominent G peak (1582 cm^−1^) in these samples, but at a relatively low intensity. According to [51], the low intensity of the G peak indicates less graphitization, which appears in samples with Q-carbon occurrence. At the same time, this can explain the increase in the intensity of the G peak in the 2000 mJ cm^−2^ sample compared to the 3000 mJ cm^−2^ sample. This statement is also confirmed by the XPS spectra shown in Section 3.2 in Figure 5. It can be seen in the spectra that the conversion of sp^2^ carbon to sp^3^ increases with increasing laser beam energy. On the other hand, a higher beam energy (2000 and 3000 mJ cm^−2^) modifies the NDs film to a greater depth, creating a Si-C bond between the NDs film and the silicon substrate. Increased oxidation also occurs in these samples, which can be reflected in the lower sp^2^ and sp^3^ hybridization content compared to other bonds. The absence of the 2160 cm^−1^ peak in the Raman spectra reflects the absence of any sp^1^ hybridization in the eventual NDs film, Q-carbon, or intermediate amorphous carbon region [11].

### 3.4. XRD Analysis

**Figure 7:**
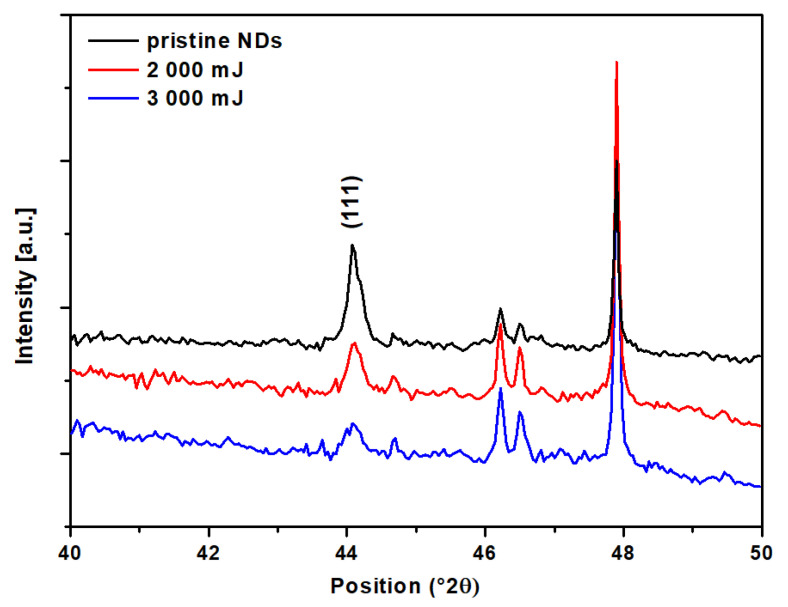
XRD diffractogram of unmodified (pristine) NDs film and NDs samples after exposure to a laser beam with energies of 2000 mJ cm^−2^ and 3000 mJ cm^−2^.

We used the XRD method to confirm the presence of diamond (111) and its modified form with Q-C. Figure 7 shows the diffractometric peaks of pristine NDs and NDs after laser annealing at energies of 2000 mJ cm^−2^ and 3000 mJ cm^−2^. Many silicon (substrate) peaks were measured when the samples were analyzed [52,53]. For this reason, we measured a narrow range of positions where the searched diamond peak and modified carbon should be located. As can be seen from Figure 7, with increasing energy there was a decrease in intensity and a broadening of the diamond peak at position 44.1°. One of the reasons may be the remelting of the original nanodiamonds, with their reclustering resulting in larger micro-/nano-diamonds. This statement agrees with the images of the surface created by SEM and AFM methods in Figure 2 and Figure 3. Other reasons (mentioned in Section 3.1) may be the micro-stress that occurs after exposure to the laser beam at the film-substrate interface, where the surface layer cracks and Q-carbon is exposed in the fibrous structure form. The broadened (111) peak may be due to defects in the carbon nanosheet during Q-carbon fabrication, which agrees with the Raman spectra in Figure 6. The remaining peaks in Figure 7 belong to the silicon substrate [29,51,54,55].

## 4. Conclusions

In our experiment, we exposed a nanocrystalline diamond film to a laser beam to investigate the changes to the irradiated sample’s surface morphology and chemical composition. NDs were exposed to laser energy values of 1600, 2000, and 3000 mJ cm^−2^. The AFM and SEM methods found that the samples were destroyed more in the NDs film with increasing beam energy to create a fibrous structure resembling Q-carbon fibers. Layered micro-/nano-spheres, representing the role of diamonds, were created at the three-point junction of the fibers. After applying the laser beam, the EDS method measured a significantly increased silicon concentration in all samples, which comprised approximately half of the total atomic concentration. This could be caused by the depth measurement of the EDS method and the partial blasting of the NDs film coated on the silicon substrate. The maximum amount of oxygen in the laser-exposed samples was 5 wt% of the total volume. The pristine NDs sample comprised 98.6% pure carbon, with a partially oxidized surface (1.0 wt%) on a silicon substrate (0.4%). Subsequently, the binding characteristics of the measured elements were determined using the XPS method. The unmodified sample (pristine NDs) consisted of 78% sp^2^ hybridized carbon, while in the laser-exposed samples, there was a significant conversion of sp^2^ to sp^3^ hybridization. As described in several articles, a Q-carbon structure (fibers) consisting of 80% sp^3^ hybridization is prepared by melting and quenching the DLC film. The samples exposed to higher energy values of the laser beam (2000 and 3000 mJ cm^−2^) had, in addition to oxygen bonds, carbide bonds characteristic of Q-carbon production. Raman spectroscopy confirmed the presence of a diamond (sp^3^) phase and a low-intensity graphitic (G) peak occurring in the Q-carbon form samples. By the XRD method we confirmed the presence of diamond (111) and its modified form with Q-C; with increasing energy, there was a decrease in intensity and a broadening of the diamond peak at position 44.1°.

## Figures and Tables

**Table 1:** Content (%) of the deconvoluted peaks in C1s spectrum in pristine NDs samples and samples with laser beam irradiation (1600, 2000, and 3000 mJ cm^−2^).

	Si-C	sp^2^	sp^3^	C-O	O-C=O	C-O-C
**pristine NDs**	-	78.4	16.8	-	-	4.8
**1600 mJ cm^−2^**	-	41.1	47.6	-	-	11.3
**2000 mJ cm^−2^**	24.8	20.7	44.0	7.6	2.9	-
**3000 mJ cm^−2^**	24.9	20.2	43.1	8.1	3.7	-

## Data Availability

Data are contained within the article.
